# Kaposi Sarcoma Mimicking Acute Flare of Ulcerative Colitis

**DOI:** 10.1177/2324709617713510

**Published:** 2017-06-12

**Authors:** Vivek Kumar, Parita Soni, Mohit Garg, Madina Abduraimova, Jonathan Harris

**Affiliations:** 1 Maimonides Medical Center, Brooklyn, NY, USA

**Keywords:** ulcerative colitis, colon cancer, Kaposi sarcoma, gastrointestinal surgery, HIV/AIDS, diagnosis, medical education

## Abstract

Besides an AIDS-defining illness, Kaposi sarcoma (KS) is also seen in individuals on long-term immunosuppressant therapy. We report KS in a 70-year-old immunocompetent man, which initially mimicked acute flare of ulcerative colitis (UC). He was hospitalized multiple times for complaints of watery diarrhea and tenesmus. Despite treatment with mesalamine, short courses of methylprednisolone, and one dose of infliximab, his symptoms improved only partially. He underwent colonoscopy, which revealed mild active colitis and a mass in the ascending colon. After treatment of acute flare with methylprednisone and mesalamine, he underwent total colectomy with end ileostomy. The histopathology confirmed stage I adenocarcinoma of colon. He continued to experience watery diarrhea, which was attributed to intractable UC, and he underwent protectomy several weeks later. The histopathology of rectum revealed KS. After surgery, watery diarrhea resolved completely. Review of literature suggests KS has been rarely reported in immunocompetent individuals with inflammatory bowel disease.

## Introduction

Kaposi sarcoma (KS) is an acquired immune deficiency syndrome (AIDS) defining illness and is associated with human herpes 8 virus (HHV-8) infection.^1^ KS is also seen commonly in patients who are on long-term immunosuppressive treatment such as after transplantation.^1^ The presentation of KS varies from isolated cutaneous lesions to visceral involvement like mouth, gastrointestinal tract, or respiratory tract with frequent overlapping. The occurrence of primary intestinal KS without cutaneous involvement is uncommon.^2^ Moreover, development of KS in patients with inflammatory bowel disease is rarely reported.^3^ We report a case of an immunocompetent patient with ulcerative colitis (UC) with chronic active proctitis who presented with intractable diarrhea for prolonged period and received multiple treatments for acute flare of UC. Due to refractory disease he underwent protectomy and was diagnosed with KS on histopathological examination of rectal tissue.

## Case Presentation

A 70-year-old man with history of hypertension and UC for 4 years presented to the emergency room with worsening of watery diarrhea, tenesmus, intermittent episodes of generalized abdominal pain, and nausea without any vomiting. He denied history of any fever, chills, anorexia, or weight loss. On initial presentation, his vital signs were stable. General physical examination revealed soft abdomen but mild tenderness was present over all quadrants with slightly increased bowel sounds. Cardiovascular, pulmonary, skin, and extremities examination was normal.

Laboratory investigations showed mild leucocytosis (white blood cells 11 400/UL), anemia (hemoglobin/hematocrit 9.9/31.8 g/dL), mild hypoalbuminemia (serum albumin 2.5 g/dL), raised erythrocyte sedimentation rate (141 mm/h), and C-reactive protein (12.74 mg/dL). Kidney, liver, and human immunodeficiency virus (HIV) panels were normal. Stool analysis revealed presence of pus cells but ova and parasites were absent. Stool culture and *Clostridium difficile* antigen tests were also negative.

Prior to this admission, the patient was hospitalized multiple times over the last several months for similar symptoms. He received multiple treatments for acute exacerbation of UC including mesalamine and short courses of methylprednisolone. He witnessed only partial improvement in the symptoms without complete resolution. For most of these episodes, he received intravenous methylprednisone during his hospital stay, and oral prednisone in tapering doses after discharge. Prior to this admission, he also underwent colonoscopies for these symptoms on 2 occasions, with the biopsy results showing chronic active inflammatory changes. The last colonoscopy was performed 8 months prior to this admission. His symptoms recurred soon after corticosteroids were stopped. Currently, he experienced worsening of symptoms despite on maintenance mesalamine therapy. He received one dose of infliximab but was discontinued due to reaction and intolerance. However, he never received any immunomodulatory drugs such as azathioprine, 6-mercaptopurine, or cyclosporine.

**Figure 1.**
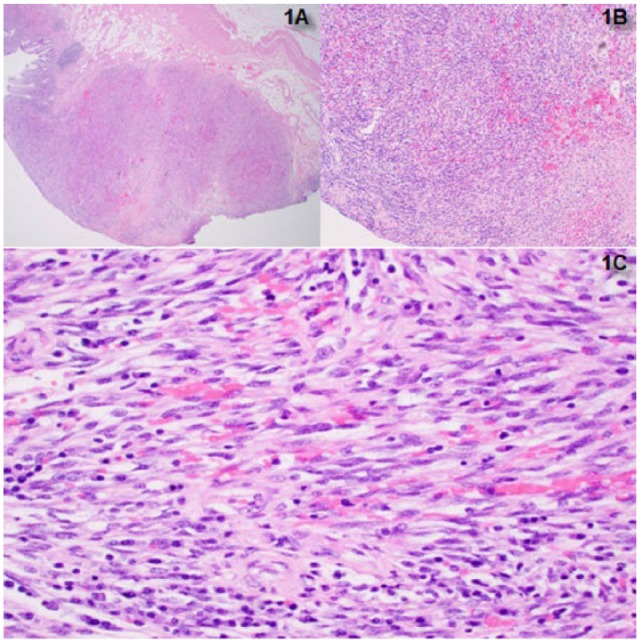
The nodular colonic mucosa in low-power view (A), medium-power view (B), and high-power view (C) showing spindle cells with admixed scattered lymphocytes and plasma cells with the presence of extravasated red blood cells.

On this admission, colonoscopy revealed a mass-like lesion in ascending colon with active colitis with marked colitis endoscopically and active chronic colitis microscopically. The biopsy of the mass showed low-grade dysplasia and focal high-grade dysplasia. As he was at a high risk of adenocarcinoma in UC and biopsy proven high-grade dysplasia, he underwent total abdominal colectomy with end ileostomy. He tolerated the surgery well and was discharged to the rehabilitation center on mesalamine and oral corticosteroids in tapering doses. The histopathology report of surgical specimen from total abdominal colectomy showed 0.3 cm adenocarcinoma arising in association with tubulovillous adenoma invading the submucosa. This mass was a tubulovillous adenoma (dysplasia seen on biopsy) with focal adenocarcinoma on resection. Proximal, distal, and mesenteric margins were negative for tumor. Pathologically, it was reported as stage pT1 tumor. Besides, histological changes consistent with chronic active UC were also present throughout the specimen (Figure 1A-C).

**Figure 2.**
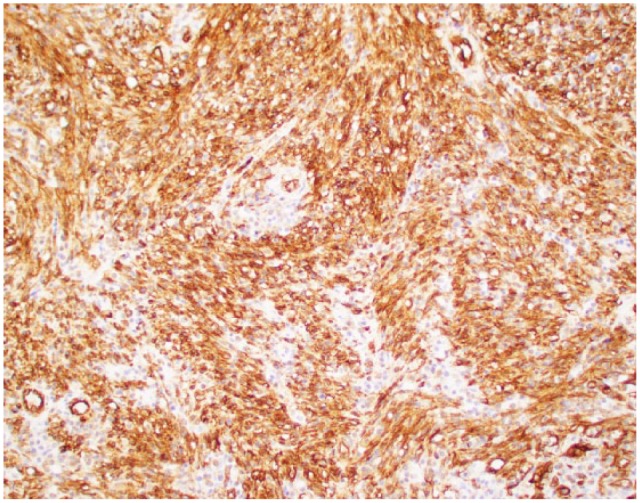
Cytoplasmic staining showing the vascular marker for CD34.

**Figure 3.**
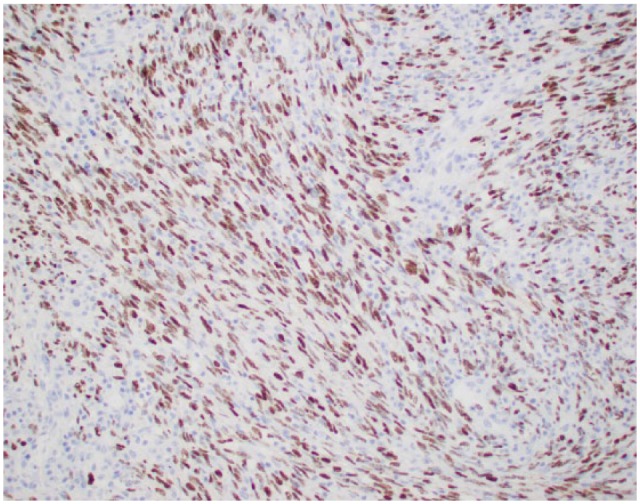
Immunohistochemistry staining showing nuclear HHV-8 positivity.

However, the patient still remained symptomatic and continued to have watery diarrhea with tenesmus. A 3-stage panproctocolectomy with creation of ileoanal J-pouch was recommended. As a first stage, patient was scheduled semi-urgently for robotic-assisted subtotal colectomy with end ileostomy. For refractory UC he underwent protectomy with ileal pouch-anal anastomosis. The biopsy of the rectal specimen from total protectomy showed up to 4 mucosal/submucosal nodules (10 mm in greatest diameter) and with 1 out of 34 lymph nodes involved by KS. On immunohistochemistry, lesional cells were positive for CD34 (Figure 2). Kappa and lambda showed a polytypic population of plasma cells in the background. The tumor cells were also positive for HHV-8 (Figure 3). Thus, the diagnosis of KS was confirmed.

The patient tolerated the procedure well. His symptoms resolved completely after surgery and is in remission currently.

## Discussion

KS is rare and it primarily affects the skin.^4^ KS is seen in following 4 forms: (1) classical, (2) AIDS-associated, (3) iatrogenic, and (4) endemic.^5^ All forms are associated with HHV-8 infection.^6-9^ The most common form seen in the United States is in association with AIDS followed by iatrogenic.^1,10^ However, the prevalence of KS has decreased in the highly active antiretroviral treatment era.

UC is a chronic inflammatory bowel disease that causes inflammation and ulcers in the digestive tract. Few case reports have been published highlighting the association of KS and UC.^1-3,6,10-13^ The clinical manifestations of KS in UC is quite variable. Most of the patients in previous case reports presented with bloody or watery diarrhea, abdominal pain, weight loss, and cachexia.^6,12-14^ Complications like severe bleeding or toxic megacolon have also been reported. Most of these patients had refractory UC requiring long-term immunosuppression.^6,12-14^ Majority of these patients also develop cutaneous lesions before or after the diagnosis of KS.^12^

This case is unique due to several reasons. First, KS was diagnosed in this patient without any underlying immunodeficiency. In the previous reports of intestinal KS with UC most of the patients were immunodeficient, either due to HIV^12^ or due to long-term immunomodulatory treatment with azathioprine or 6-mercaptopurine.^13^ This patient received corticosteroids intermittently but most likely KS developed prior to these exacerbations.

Second, the diagnosis of primary gastrointestinal KS in the absence of any skin lesions is difficult to make.^12^ Most of the patients in prior case reports developed cutaneous forms of KS during the course of illness. This patient did not have any skin lesions suspicious of KS.

Third, this patient underwent colonoscopy multiple times but did not have any lesions with appearance suspicious of KS. Most of the patients reported in the past had colonic nodular lesions that raised suspicion of KS and was later confirmed on biopsy. Moreover, the coexistence of adenocarcinoma, KS, and UC has not been described previously.

The concurrent diagnosis of KS in UC patients without underlying immunodeficiency is the matter of active research.^15^ In vitro studies have highlighted the possible carcinogenic effect of immunomodulatory drugs.^16^ However, clinical studies have failed to show any causal effect of immunomodulatory drugs on development of KS.^16^ UC is a well-known precursor of colonic adenocarcinoma; however, its role in the genesis of KS is unlikely and has not been proven.^17^

## Conclusion

Kaposi sarcoma can be seen in ulcerative colitis patients without any underlying immunodeficiency or in the absence of long-term immunosuppressive treatment. The clinical manifestations of Kaposi sarcoma in ulcerative colitis is variable and may be confused with acute exacerbation of ulcerative colitis often delaying the diagnosis. In the patients with ulcerative colitis, Kaposi sarcoma can affect only gastrointestinal tract in the absence of skin lesions.
